# Gradient-Modified HfC-SiC Mixed Bi-Interlayers Synthesized under Different TMS Flow Rate Increment for Depositing Diamond Coating onto WC-Co Substrate

**DOI:** 10.3390/ma13071558

**Published:** 2020-03-27

**Authors:** Ke Zheng, Jie Gao, Shengwang Yu, Yongsheng Wang, Hongjun Hei, Yong Ma, Bing Zhou, Bin Tang, Yucheng Wu

**Affiliations:** 1 Institute of New Carbon Materials, Taiyuan University of Technology, Taiyuan 030024, China; zhengke0401@163.com (K.Z.); yushengwang@tyut.edu.cn (S.Y.); wangyongsheng@tyut.edu.cn (Y.W.); heihongjun@tyut.edu.cn (H.H.); mayong@tyut.edu.cn (Y.M.); zhoubing@tyut.edu.cn (B.Z.); wyc@tyut.edu.cn (Y.W.); 2 National Key Laboratory of Science and Technology on Vacuum Electronics, School of Electronic Science and Engineering, University of Electronic Science and Technology of China, Chengdu 610054, China

**Keywords:** HfC-SiC mixed bi-interlayers, TMS flow rate increment, gradient-modified structure, adhesion, hardness, diamond coating

## Abstract

To deposit well-adhered diamond coating, gradient-modified hafnium carbide-silicon carbide (HfC-SiC) mixed bi-interlayers were prepared on cemented carbides (WC-Co) by plasma surface metallurgy technique under the different tetramethylsiline (TMS) flow rate increment. The effects of the TMS flow rate increment on the composition, microstructure, adhesion, and hardness of the bi-interlayers were investigated. Then, the well-adhered bi-interlayer was chosen for the deposition of the diamond coating. It was found that the HfC-SiC mixed bi-interlayers consisted of a diffusion-modified HfC-riched inner layer and a SiC-riched outer layer. The TMS flow rate increment played a key role in tailoring the surface morphology, thickness, and interface character of the bi-interlayer. The dense nanocrystalline diamond coating was formed on the optimized bi-interlayer at the increment of 0.20 sccm/2 min. The diamond coating showed excellent adhesion, which was benefited from the cobalt (Co) diffusion inhibition, gradient composition distribution, and mechanical interlocking.

## 1. Introduction

Diamond-coated cemented carbides (WC-Co) cutting tools have shown great potential in machining non-ferrous metals and their alloys, particulate or fiber-reinforced composite materials, and ceramic composites due to the excellent wear resistance of diamond [1,2]. Unfortunately, the actual lifetime is lesser than expected because of the insufficient coating adhesion. Cobalt (Co) binder in the WC-Co substrate deteriorates the coating adhesion by inhibiting diamond nucleation and promoting the formation of graphitic phases at the interface [3,4]. Moreover, the residual stress caused by the mismatched thermal expansion coefficient (TEC), Young’s modulus, and hardness, etc. between the WC-Co substrate and the diamond coating results in an increased risk of the coating delamination under the shear force during the cutting application [5,6].

In order to suppress the adverse effect of Co binder and improve adhesion, one simple and cheap approach in industrial production is removing Co binder from the WC-Co surface by chemical etching [7]. However, this method always worsens the mechanical strength of the WC-Co substrates [8]. An alternative way is depositing an interlayer on the WC-Co surface as a diffusion barrier [6]. Owing to the effective prevention of Co diffusion and reduction of interfacial residual stress, up to now, various intermediate layers have been fabricated successfully. They mainly include single-interlayers, such as Ta [9], Cr [9], Al [10], TiC [11], TiN [12], CrN [13], and β-SiC [14,15], and multi-interlayers, such as Cr/CrN [5], W/Al [16], Cr_2_O_3_/Cr [17], Al/AlN [18], and Mo/[TaN/ZrN]_9_ [19]. Generally, the single-interlayers cannot simultaneously satisfy the requirements of inhibiting cobalt diffusion, mitigating TEC mismatch, and enhancing adhesion and nucleation rate of the diamond coating. By comparison, multi-interlayers can realize the requirements by its different layers. Polini et al. [5] prepared a CrN inner layer as a diffusion barrier and TEC buffer layer and Cr outer layer as an adhesion enhancing layer. Ye et al. [17] deposited Cr_2_O_3_ inner layer as the diffusion layer and Cr outer layer as an adhesion enhancing layer. Poulon-Quintin et al. [19] deposited the TaN single layer or TaN/ZrN multilayer as Co diffusion barrier and Mo outer layer as the nucleation layer. Because of reasonable designations of the above research works, the diamond coatings on their interlayers possess good adhesion. Nevertheless, multi-interlayer means multi-interfaces, which are detrimental to the adhesion properties for the interlayer itself and the subsequently deposited diamond coating. Therefore, the adhesion properties of the multi-interlayer system should be further improved. Some researchers have fabricated gradient-modified interlayers in order to reduce interfaces and adjusting TEC mismatch [20,21,22]. The drawback is the gradient structure only exists in the interlayer, while a non-gradient interface still exists between the interlayer and the substrate.

Plasma surface metallurgy technique (PSMT) is an effective approach to prepare not only the deposited layer but also the diffused layer on the substrate [23]. The surface of the heated substrate is bombarded by the plasma, leading to the enhanced diffusion. As a result, the composition distributes gradually between the coating and the substrate, resulting in the well-matched TECs and good adhesion strength [24]. In our previous investigations, we prepared Mo/Mo_2_C interlayers [25], silicon carbide/hafnium carbide (SiC/HfC) dual-interlayer [26], and HfC-SiC/HfC dual-interlayer [27] on the WC-Co substrate by PSMT for the diamond deposition. Those interlayers could, effectively, prevent the outward diffusion of Co. What is more, the thick diffusion layer was formed at the interface between the interlayer and the WC-Co substrate. Hence, diamond coatings with improved adhesion strength were obtained. Compared with the Mo/Mo_2_C interlayers, the SiC/HfC dual-interlayer and the HfC-SiC/HfC dual-interlayer both have two layers. The inner HfC layer can prevent Co diffusion, and the outer SiC-dominant layer or gradient HfC-SiC layer can mitigate TEC mismatch. That’s why the diamond coatings on the dual-interlayers have better adhesion strength. Between the two types of dual-interlayers, the HfC-SiC/HfC dual-interlayer has gradient composition distribution at the inner/outer layer interface. Such a gradient interfacial structure is beneficial to manipulate the TEC mismatch and avoid introducing extra interfacial thermal stress. As a result, the diamond coating on the HfC-SiC/HfC dual-interlayer has better adhesion than that on the SiC/HfC dual-interlayer.

In this paper, as an extension of previous, HfC-SiC mixed bi-interlayers with different gradient composition distribution at the inner/outer layer interface were prepared onto WC-Co substrates through manipulating the tetramethylsiline (TMS) flow rate with different increment. The effects of the TMS flow rate increment on the microstructure, adhesion, and hardness of the bi-interlayers were investigated. Thereafter, the optimized bi-interlayer at the increment of 0.20 sccm/2 min was employed as the intermediate layer to deposit the diamond coating. Finally, the adhesion property of the coating system was evaluated, and the adhesion enhancement mechanism was analyzed.

## 2. Experimental Procedure

### 2.1. Preparation of the HfC-SiC Mixed Bi-Interlayers

**Figure 1:**
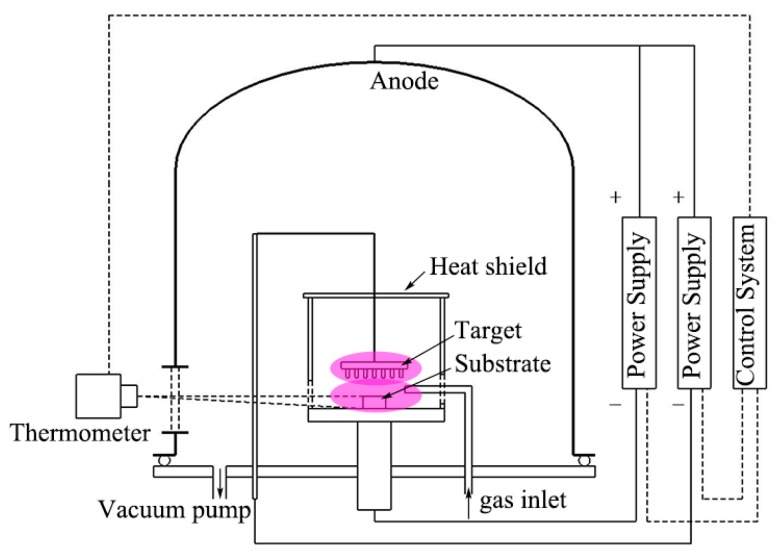
The schematic diagram of the plasma surface metallurgy technique (PSMT) furnace.

**Figure 2:**
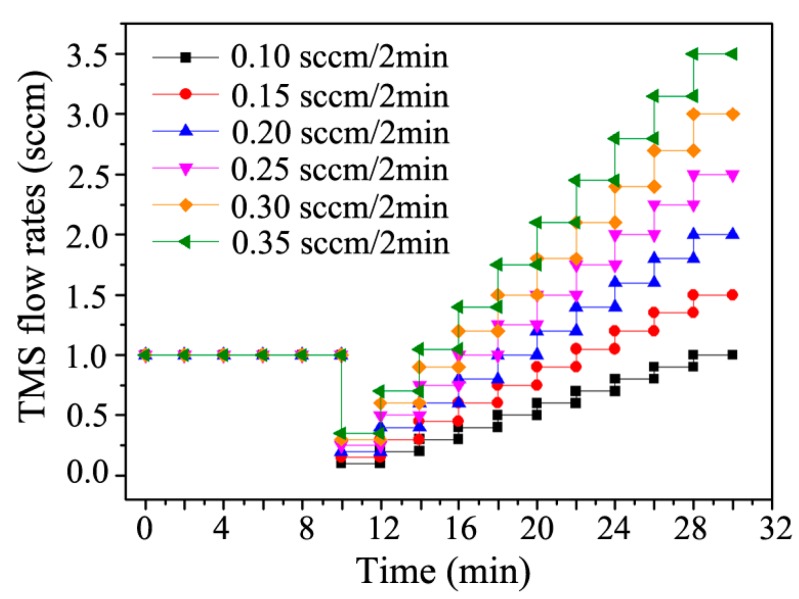
TMS (tetramethylsiline) flow rates during the second and third steps in the PSMT treatment as a function of time.

Commercial WC-6 wt. % Co sheets with a size of 10 mm × 10 mm × 4 mm were used as substrates in this study. The HfC-SiC mixed bi-interlayers were synthesized in a PSMT furnace [23]. The schematic diagram of the furnace is shown in Figure 1. Two cathodes composed of a substrate electrode and a target electrode are installed in the furnace chamber, which is used as an anode. Once the chamber is evacuated, argon (Ar) is put in, and the two cathode powers are turned on, and the two groups of glow discharge will generate around the target and the substrate. One glow discharge heats the substrate to an expected temperature, while the other bombards the target to generate target ions, atoms, and particles. Then, the sputtered ions, atoms, and particles will deposit onto the substrate directly or react with reactive gas and deposit onto the substrate. In our experiment, a special target was used, which was a graphite plate inlaid with 99.9 wt % Hf rods. The distance between the substrate and the Hf rods was 15 ± 2 mm. Three types of gas hydrogen (H_2_) with a purity of 99.99%, TMS with a purity of 99.99%, and Ar with a purity of 99.99% were adopted during the PSMT process. Here, TMS and H_2_ were used as precursors for forming HfC and SiC, and Ar was applied as a protective and plasma excitation gas. A continuous three-step process was adopted to obtain the HfC-SiC mixed bi-interlayers. Firstly, Ar^+^ sputtering was actualized to clean and activate the surface of WC-Co by introducing Ar in the furnace with a flow rate of 70 sccm. Secondly, TMS with a flow rate of 1 sccm was added in the furnace to deposit the inner HfC-SiC mixed layer. Thirdly, H_2_ with a flow rate of 10 sccm was also added together with gradually increased TMS to obtain the outer HfC-SiC mixed layer. The TMS flow rate adopted at the second and third steps as a function of time is shown in Figure 2. It could be observed that the TMS flow rates increased like a ladder in the third step. The increment (refer to the TMS flow rate increment per 2 min) varied with different ultimate TMS flow rates. Other process parameters were conducted under the following conditions: gas pressure 35–64 Pa, source electrode voltage −720~−780 V, substrate voltage −470~−530 V, voltage difference 250 V, substrate temperature 800 ± 5 °C.

### 2.2. Deposition of Diamond Coating

A self-developed microwave plasma chemical vapor deposition (MPCVD) system was used to deposit a diamond coating on the HfC-SiC mixed bi-interlayer-coated WC-Co substrate [28]. Before the diamond deposition, the bi-interlayer-coated WC-Co substrate was firstly treated by an ultrasonic concussion for 30 min in an acetone suspension containing diamond powders. Subsequently, another ultrasonic concussion in an ethanol bath was adopted to clean the samples. During the deposition, a gas mixture of H_2_, CH_4_, and Ar was used for diamond synthesis, whose flow rates were 30 sccm, 5 sccm, and 120 sccm, respectively. The microwave power was kept at 1000 W, the gas pressure in the chamber was ~12 kPa, the temperature of the bi-interlayered WC-Co substrate was maintained at 800 °C ± 10 °C, and the deposition time was 4 h. 

### 2.3. Characteristics of HfC-SiC Mixed Bi-Interlayer and Diamond Coating

The phase structures of the HfC-SiC mixed bi-interlayers and the diamond coating were studied by means of X-ray diffraction (XRD, DX-2700 X) with Cu Kα radiation. Surface and cross-sectional microstructure and elemental distributions were examined by a field emission scanning electron microscopy (FESEM, TESCAN MIRA3 LMH) equipped with X-ray energy dispersive spectrometry (EDS) detector. The surface roughness of the bi-interlayers was measured by atomic force microscopy (AFM, SEIKO SPA-300HV). The quality of the diamond phase was assessed by using a Raman spectrometer (Renishaw in Via-Reflex), in which the 532 nm green line of a 25 mW Nd:YVO_4_ DPSS laser was adopted. 

### 2.4. Mechanical Properties of HfC-SiC Mixed Bi-Interlayer and Diamond Coating

A Vickers microhardness tester (LECO M-400-H1) was used to measure the surface microhardness of the original substrate and the interlayers. In order to reduce experimental error, five test points were randomly chosen. A loading force of 0.98 N was used to reduce the substrate’s effect. Besides, the maximum load was held for 20 s to reduce the creep deformation. An HT-5001 scratch tester was adopted to estimate the adhesion of the bi-interlayers. During the test, a linearly increasing load from 5 N to 180 N was applied to a spherical Rockwell C diamond indenter at a loading rate of 100 N/min. The indenter slid over the interlayer surface at a speed of 2 mm/min. At the same time, the acoustic emission (AE) signal was recorded and displayed as a function of load. A Rockwell C indentation tester was applied to evaluate the adhesion properties of the diamond coating with a load of 1470 N. The Verein Deutscher Ingenieure 3198 norm (VDI 3198) was used to classify the adhesion quality [29]. 

## 3. Results and Discussion

### 3.1. The Microstructure of the Prepared HfC-SiC Mixed Bi-Interlayers

**Figure 3:**
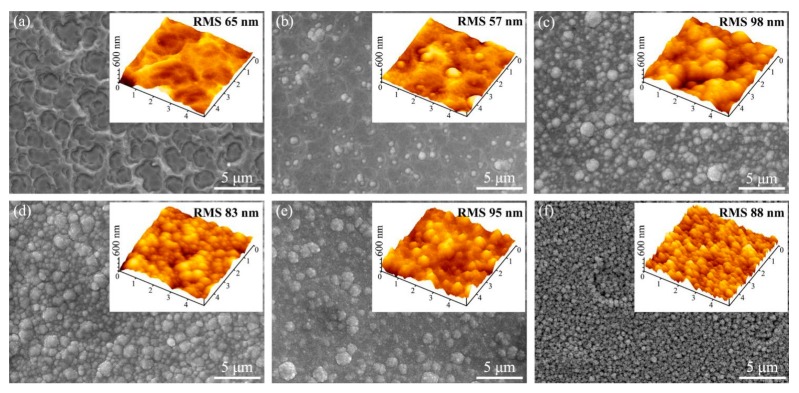
SEM and AFM images of the hafnium carbide-silicon carbide (HfC-SiC) mixed bi-interlayers deposited with different TMS flow rate increment: (**a**) 0.10 sccm/2 min, (**b**) 0.15 sccm/2 min, (**c**) 0.20 sccm/2 min, (**d**) 0.25 sccm/2 min, (**e**) 0.30 sccm/2 min, (**f**) 0.35 sccm/2 min.

Figure 3 presents the surface microstructure of the as-prepared bi-interlayers. For the bi-interlayer prepared at the TMS flow rate increment of 0.1 sccm/2 min, interactional annular morphologies could be found on the whole surface (Figure 3a). From the inserted AFM image, the annular structure was a pit with a rough interior. When the increment increased to 0.15 sccm/2 min (Figure 3b), some small bulged clusters scattered on the surface. With a further increase in the increment, the quantity and diameter of bulged clusters both increased steadily. The clusters distributed on the whole surface corresponding to increment above 0.25 sccm/2 min. It should be noted that, when the increment was 0.35 sccm/2 min, the surface was composed of small-sized loose clusters, and voids could be observed at cluster interspaces. According to the research of Rong et al. [30], point discharges are generated by the effect of hollow cathode discharge and the special design of the Hf target. As a consequence, the sputtered atoms/ions collided on the interlayer surface with different strength, which caused the annular pit morphologies. With the increase of the TMS flow rate increment, the concentration fluctuation of TMS became more intense, which brought about the formation of bulged clusters at the high concentration sites. The higher the increment was, the more the high concentration sites were. Hence, the number of bulged clusters increased with the increase of the TMS flow rate increment, which led to surface morphology variations.

The rough interlayer surface with micro-defects can promote the diamond nucleation by reducing the critical nucleation energy and enhance adhesion by increasing the mechanical interlocking force [31,32]. The root mean squared roughness of the bi-interlayer surface over an area of 100 m^2^ is given in the inserted AFM images. No obvious regularities could be found. Roughly, the bi-interlayers with bulge-shaped morphology had higher surface roughness than that with pit-shaped morphology. The bi-interlayer prepared at an increment of 0.20 sccm/2 min possessed the highest roughness of 98 nm.

**Figure 4:**
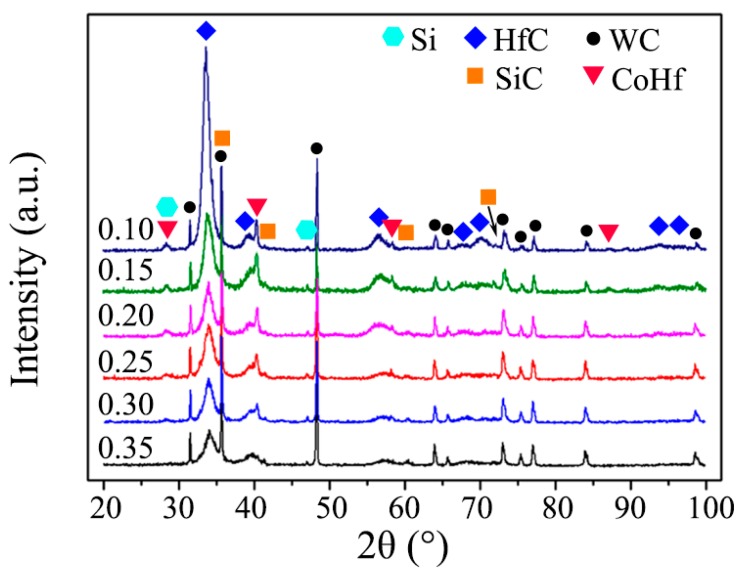
XRD patterns of the bi-interlayers deposited with different TMS flow rate increment.

Figure 4 shows the XRD patterns of the bi-interlayered specimens. The broad HfC peaks, weak SiC, Si, and CoHf peaks, as well as strong and sharp WC peaks, could be detected. Wherein, the intensity of the HfC and CoHf peaks decreased with increasing increment. The appearance of HfC was due to the chemical reaction between the sputtering Hf from the target and the decomposed C from the introduced TMS. TMS was also the cause of SiC and Si phases. The emergence of CoHf peaks demonstrated that interfacial chemical reaction happened at the interface between the interlayer and the WC-Co substrate. The intensity variation of HfC was related to the increasing increment, which led to an increase of the SiC content but a decrease of HfC content.

Here, the broad HfC peaks could also be observed in all the XRD patterns, indicating the fine polycrystalline grains. Using the Debye–Scherrer’s equation [33], the collective mean crystallite size of HfC could be estimated. The measured full width at half maximum *β* of HfC (111) plane diffraction and the estimated crystallite size *D* are listed in Table 1. The size of the nanoscale HfC reduced with increasing increment. A possible reason was that the SiC phase of the interlayer inhibits the growth of the HfC grain as the second phase [34].

**Figure 5:**
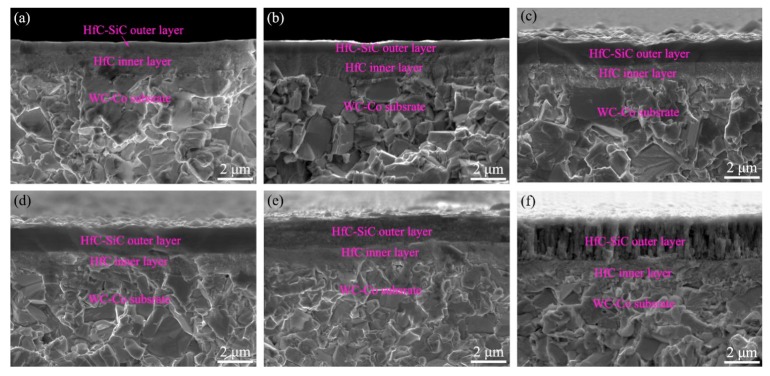
Cross-sectional SEM images of the HfC-SiC mixed bi-interlayers prepared with different TMS flow rate increment: (**a**) 0.10 sccm/2 min, (**b**) 0.15 sccm/2 min, (**c**) 0.20 sccm/2 min, (**d**) 0.25 sccm/2 min, (**e**) 0.30 sccm/2 min, (**f**) 0.35 sccm/2 min.

Figure 5 depicts the cross-sectional microstructure of the HfC-SiC mixed bi-interlayers. All the cross-sections included an inner layer of nanocrystalline structure and an outer layer of smooth and compact structure. Interfaces of gradient variation could be observed between inner and outer layers for the bi-interlayers synthesized at a low increment of 0.10 and 0.15 sccm/2 min. But the interface became more and more obvious with increasing increment. Besides, some microstructure changes took place in the outer layer of the samples prepared at an increment of 0.30 and 0.35 sccm/2 min (Figure 5e,f). Both the outer layers had loose structures. Especially, cavities could be clearly observed in the specimen prepared at 0.35 sccm/2 min. 

**Figure 6:**
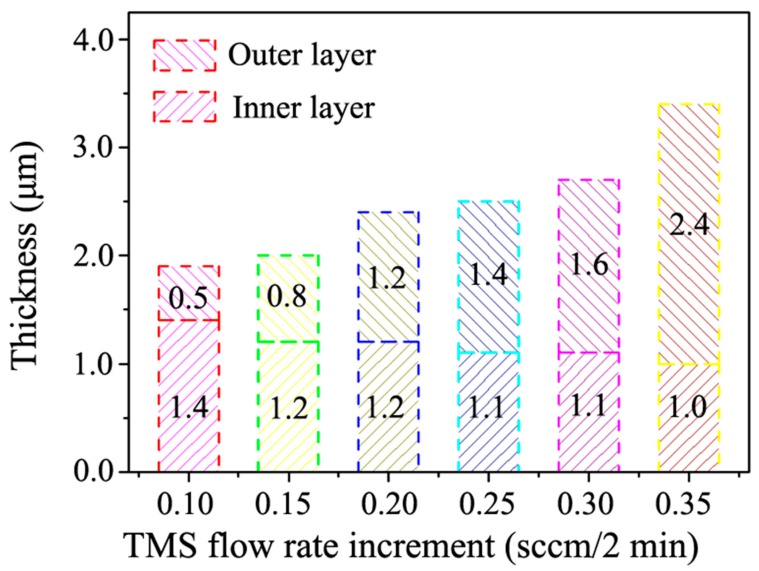
Variation in thickness of HfC-SiC mixed bi-interlayers as a function of TMS flow rate increment.

In addition to the microstructure variations, the thickness of the HfC-SiC mixed bi-interlayers also changed with the TMS flow rate increment. The thickness of the inner layers, outer layers, and the whole interlayers as a function of the increment are plotted in Figure 6. The thickness decreased for the inner layers but increased for the outer layers with the increase of the increment. Due to the benefit from more significant changes of the later, the whole thickness of the whole interlayers increased gradually. 

The gradually decreasing thickness of the inner layers and the resulting obvious inner/outer layer interface could be ascribed to the ever-increasing TMS flow rate during the PSMT process. In the third step, it took a certain period for TMS and H_2_ to reach a high enough concentration to form the smooth outer layer. During this period, the synthesis of SiC and HfC was performed simultaneously, but the content of HfC decreased gradually, while that of SiC increased constantly. The mentioned time would be shortened because of the increase of the increment, leading to the decrease of the actual deposition time of the inner layer and its thickness. Besides, the shortened time also led to the increasing concentration gradient of HfC and SiC, causing a sharper and sharper inner/outer layer interface. The ever-increasing increment also promoted the synthesis of SiC and, thus, led to the thickness increase of the outer layers. However, high increment (0.30 and 0.35 sccm/2 min) might bring about excessive TMS concentration fluctuation at the growth front of the bi-interlayers, influencing the growth of the SiC phase and eventually resulting in the loose structure of the outer layer [35].

**Figure 7:**
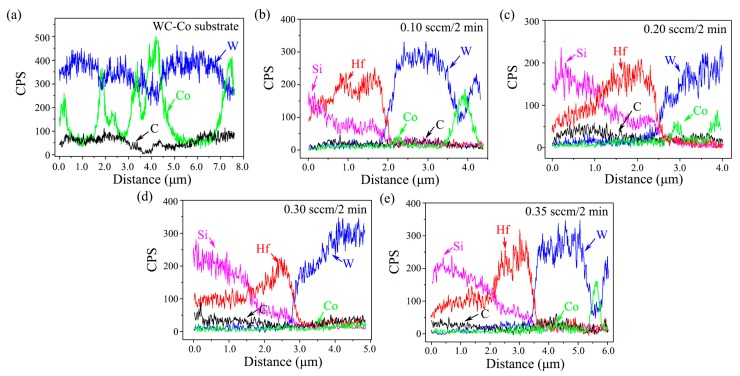
Typical EDS patterns of (**a**) WC-Co substrate and HfC-SiC mixed bi-interlayers prepared with TMS flow rate increment of (**b**) 0.10 sccm/2 min, (**c**) 0.20 sccm/2 min, (**d**) 0.30 sccm/2 min, and (**e**) 0.35 sccm/2 min.

Figure 7 shows the typical EDS line scans of the elements in the substrate and bi-interlayered specimens. In Figure 7a, the signal of three substrate elements—W, Co, and C—was present in the original substrate. The signal of W and Co showed an alternate intensity change because the Co binder only existed on the gap between tungsten carbide particles. In Figure 7b–e, besides the W, Co, and C elements, the signal of Hf and Si was also present in the bi-interlayered specimens. The signal of Hf and Si appeared in the bi-interlayer region, showing an opposite trend, where Hf increased gradually, while Si decreased gradually. The relative signal intensity of Si was higher than that of Hf in the outer layer region, but the opposite trend was present in the inner layer region. The signal intersection of Hf and Si became deeper from the surface with increasing increment; moreover, the signal gradient near the intersection got even steeper. Gradient distribution of elements could also be found at the interface between the bi-interlayer and the substrate, indicating the formation of a diffusion layer. Combining the XRD and SEM results, it could be concluded that: (1) HfC and SiC distributed gradually in the whole bi-interlayer; (2) the outer layer was a SiC-riched HfC-SiC mixed layer, while the inner layer was HfC-riched HfC-SiC mixed layer; (3) a diffusion layer was formed at interlayer/substrate interface; (4) the concentration gradient of HfC and SiC at the inner/outer layer interface became even steeper with increasing increment. Both the gradient-modified inner/outer layer interfaces and the diffusion-modified interlayer/substrate interfaces were conducive to reduce the mismatch of TECs and enhance the interfacial adhesion. In addition, we could conclude that the outward diffusion of Co had been inhibited effectively because no signal of it existed in the interlayer regions.

### 3.2. Adhesion and Surface Microhardness of the Bi-Interlayers

**Figure 8:**
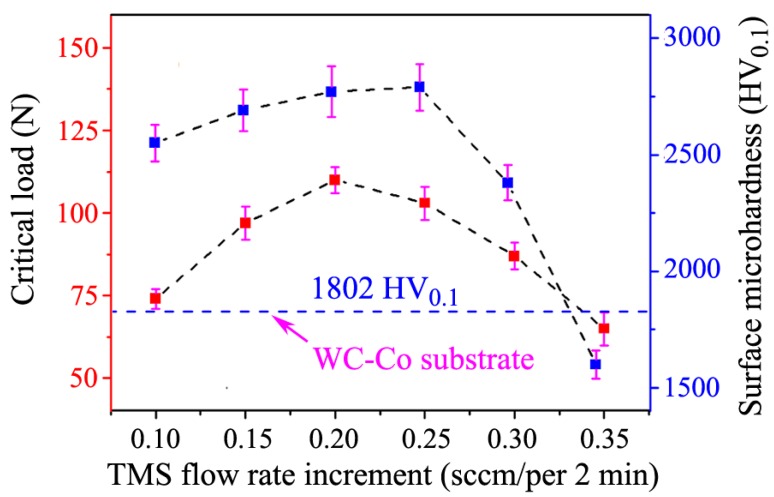
Critical load for coating delamination and surface microhardness of the HfC-SiC mixed bi-interlayers as a function of TMS flow rate increment.

The adhesion strength and surface microhardness of the HfC-SiC mixed bi-interlayers were evaluated by scratch tests and Vickers microhardness tests, as shown in Figure 8. Both the critical load for coating delamination from substrates and the surface microhardness increased firstly and then decreased with increasing increment. The maximum critical load and surface microhardness were 110 N at the increment of 0.20 sccm/2 min and 2790 HV_0.1_ at the increment of 0.25 sccm/2 min, respectively. The maximum microhardness was close to the hardness of SiC in theory (3000 HV) [6].

Both adhesion and hardness variations could be attributed to the introduction of SiC in the bi-interlayers and the microstructure of bi-interlayers. SiC has lower theoretical TEC and higher theoretical hardness than those of HfC and WC-Co [6,36]. Therefore, SiC with gradient distribution in the bi-interlayers was helpful in tailoring the TEC between the interlayers and the substrates. Moreover, the ever-increasing content of SiC with increasing increment led to the rising adhesion and surface microhardness. However, the loose microstructures and cavities (Figure 5e,f) weakened the hardness of the bi-interlayers [37]. Moreover, these cavities would act as crack-nucleation sites, resulting in the formation of cracks in the interlayer, thus deteriorating the adhesion property [38]. Besides, the resulting sharper inner/outer layer interface would reduce the beneficial effect of gradient structure on TEC regulation, leading to the adhesion decrease. Therefore, the HfC-SiC mixed bi-interlayers prepared at 0.20 and 0.25 sccm/2 min had better adhesion and microhardness properties than others. Furthermore, the adhesion and the microhardness at these two parameters were very close to each other. Due to the rougher surface, the bi-interlayer prepared at the increment of 0.20 sccm/2 min was chosen for the diamond deposition.

### 3.3. Deposition of Diamond Coatings on Well-Adhered HfC-SiC Mixed Bi-Interlayers 

**Figure 9:**
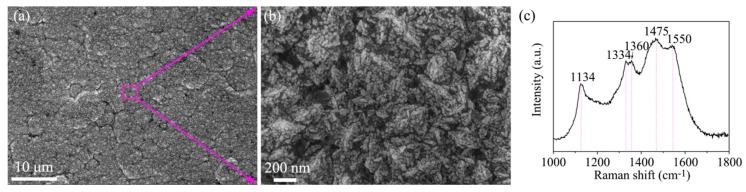
(**a**,**b**) SEM images and (**c**) Raman spectra of as-deposited diamond coatings on the HfC-SiC bi-interlayered substrates; (**b**) shows the partial enlargements of the overall views in (**a**).

Figure 9 shows the surface morphology of the as-deposited diamond coating on the HfC-SiC mixed bi-interlayer and corresponding Raman spectrum. In Figure 9a, a uniform and dense diamond coating composed of clusters—the so-called cauliflower-like diamond, could be observed. In the magnification image, the clusters consisted of accumulated granular nanocrystals. A typical Raman spectrum of nanocrystalline diamonds could be observed in Figure 9c. The diamond peak presented at ~1334 cm^−1^. The broad peak width was due to the fine crystallite size of the diamond [14]. The peaks at ~1134 cm^−1^ and ~1475 cm^−1^ were the transpoly-acetylene (Trans-PA), which are often used as the fingerprint of nanocrystalline diamond [21]. D-band at 1360 cm^−1^ and G-band at 1550 cm^−1^ are related to amorphous carbon [12].

In addition, the diamond peak shifted to higher wavenumbers from the standard diamond peak at 1332 cm^−1^, demonstrating compressive stress inside the coating [17]. This was because diamond possesses lower TEC than the interlayer and the WC-Co substrate, which leads to the formation of compressive stress in the diamond coating during cooling down [26]. The residual stress in the deposited coating could be evaluated through the equation $\sigma =-0.567\left(v_{m}-v_{0}\right)\;$, where $v_{m}$ is the measured diamond peak position, and $v_{0}$ is 1332 cm^−1^ [39]. In our diamond coating, the residual stress was calculated to be −1.7 GPa. The presence of compressive stresses could blunt crack tips and inhibit crack propagation in the diamond coating under tensile loading conditions [40,41]. From this point of view, the compressive residual stress is beneficial to the adhesion of diamond coatings, especially compared to tension residual stress. Nevertheless, the large compressive residual stress also reduces the adhesion of the diamond coating [39]. From Equation (2), it was obvious that stress was positively correlated with the Raman shift. The larger the Raman shift, the bigger the compressive residual stress. 

**Figure 10:**
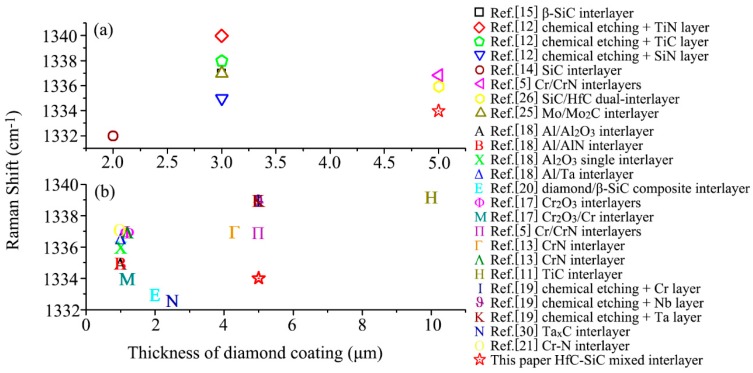
Raman shift of (**a**) nano- and (**b**) micro-diamond coatings deposited on WC-Co substrates with different interlayers.

Figure 10 shows our gathered Raman shifts of diamond coatings on different interlayers-coated WC-Co substrates. Because the coating thickness and interlayer types are the main factors influencing the coating stress, they were the focus of comparison. Figure 10a displays the Raman shift of nano-diamond coatings. It could be observed that our nano-diamond coating had the minimum Raman shift, except for diamond coating with a thickness of 2 μm deposited on SiC interlayer. Especially, the Raman shift in this paper was smaller than those of our previous reports on nano-diamond coatings on SiC/HfC dual-interlayer and Mo/Mo_2_C interlayer, indicating an improvement in decreasing residual stress. In Figure 10b, except for thin micro-diamond coatings on the TaxC interlayer [30] and diamond/*β*-SiC composite interlayer [20], our nano-diamond coating also had lower Raman shift/residual stress than micro-diamond coatings on other interlayers. To sum up, our gradient- and diffusion-modified HfC-SiC mixed bi-interlayer was a good candidate for the deposition of diamond coating with low residual stress.

**Figure 11:**
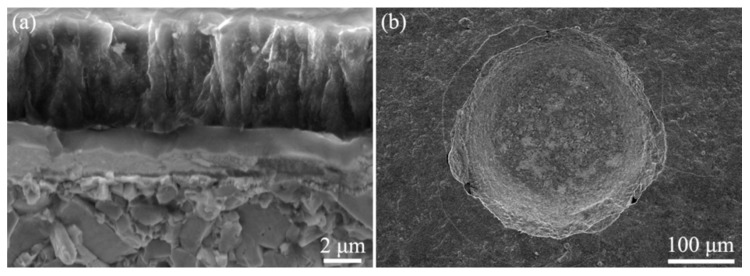
(**a**) Cross-sectional morphology of the diamond/HfC-SiC multi-coated substrate and (**b**) Rockwell C indentation on it.

Figure 11 depicts the cross-sectional microstructure of the diamond coating on the HfC-SiC mixed bi-interlayer and Rockwell C indentation made on it. The thickness of the diamond coating was about 5 μm. Its cross-section showed a structure similar to the columnar crystal (Figure 11a). However, its details were composed of fine nanocrystals, affirming the nanocrystalline diamond coating. In Figure 11b, only several annular cracks could be observed around the indentation, indicating the good adhesion of the coating system. According to the VDI 3198, the adhesion refers to class HF1~HF2. The adhesion rank of the diamond coating on HfC-SiC mixed bi-interlayer reflected by Rockwell C indentation test at a load of 1470 N was about the same as that of diamond coatings on *β*-SiC interlayer [14,15], Al/AlN interlayer [18], and Ta_x_C interlayer [30], while the diamond coating here was relatively thick. In addition, the coating adhesion was stronger than that of diamond coatings on SiC/HfC dual-interlayer [26], Mo/Mo_2_C interlayer [25], prepared by ourselves, and some other metal-based [11,18], oxide-based [17,18] interlayers, prepared by other researchers, no matter their coating thickness was thicker or thinner. The adhesion result was consistent with the residual stress (estimated by Raman shift, as shown in Figure 10), that is, the smaller the Raman shift/residual stress, the better the adhesion. Taking into account the coating thickness, it could be concluded that the diamond coating on the HfC-SiC mixed bi-interlayer had better adhesion than that on the above-compared interlayers. 

Here, the HfC-riched inner layer realized one main function of the interlayer, inhibiting the outward diffusion of Co. This could be attributed to the formation of the CoHf phase and the dense structure of the bi-interlayer, especially the nanostructure of the HfC-riched inner layer. Tang et al. [42] pointed out that forming stable cobalt compounds could deactivate Co, thereby inhibiting its outward diffusion. Poulon-Quintin et al. [19] indicated that increased grain boundaries could increase diffusion path length. Therefore, both the formation of CoHf and the fine nanocrystalline of the HfC-riched inner layer played a key role in inhibiting the diffusion of Co.

The reasonable microstructure of the HfC-SiC mixed bi-interlayer realized another main function of the interlayer, cutting down the residual thermal stress in the diamond/HfC-SiC multi-coated system. Based on the microstructure analysis, we knew that the application of the PSMT and continuously increased TMS flow rate would bring about gradient evolution of composition in the whole interlayer and at the interface between interlayer and substrate. Besides, the ever-increasing content of the SiC phase in the bi-interlayer was helpful in tailoring the TEC and hardness to suit the diamond coating. As a result, the hardness from the WC-Co substrate to the diamond increased gradually, while the TEC firstly increased and then decreased. Such a gradual evolution of composition, TEC, and hardness is beneficial for the reduction of residual thermal stress [43]. In addition, the rough surface of the bi-interlayer realizes the function of promoting diamond nucleation and improving coating adhesion by enhancing the mechanical interlocking between the diamond coating and the interlayer [44]. Under the combined effect of the above benefits, the diamond coating with excellent adhesion strength and matched performance was finally obtained.

## 4. Conclusions

In this paper, gradient-modified HfC-SiC mixed bi-interlayers were fabricated onto WC-Co substrates by continuously increasing the TMS flow rate. The bi-interlayers were composed of an inner HfC-riched layer and an outer SiC-riched layer. Both HfC and SiC distributed as a gradual approach toward each other within the whole bi-interlayer. The TMS flow rate increment played a key role in the growth of the HfC-SiC mixed bi-interlayers. The surface morphology of the bi-interlayers evolved from pits to bulge-shaped morphology with increasing increment. The thickness increased for the outer layer but decreased for the inner layer. The inner/outer layer interface became sharper and sharper with the increasing increment. The bi-interlayer prepared at the increment of 0.20 sccm/2 min displayed better mechanical properties, including adhesion strength, hardness, and surface roughness, which was employed as the intermediate layer to deposit the diamond coating. Finally, the diamond coating with excellent adhesion (HF1~HF2) was obtained. The improved adhesion for this coating system was attributed to the inhibited diffusion of Co by the bi-interlayer, the reduced residual thermal stress due to the gradient composition distribution in the whole interlayer, and the increased mechanical interlocking force owing to the rough surface.

## Figures and Tables

**Table 1:** The β-D parameters of hafnium carbide (HfC) (111) plane diffraction vs. different tetramethylsiline (TMS) flow rate increment.

TMS Flow Rate Increment	HfC(111)	
	β(^o^)	D(nm)
0.10 sccm/2min	1.13	7.3
0.15 sccm/2min	1.31	6.3
0.20 sccm/2min	1.45	5.7
0.25 sccm/2min	1.67	4.9
0.30 sccm/2min	1.70	4.8
0.35 sccm/2min	1.79	4.6
